# Data Clustering using Memristor Networks

**DOI:** 10.1038/srep10492

**Published:** 2015-05-28

**Authors:** Shinhyun Choi, Patrick Sheridan, Wei D. Lu

**Affiliations:** 1 Department of Electrical Engineering and Computer Science, University of Michigan, Ann Arbor, Michigan 48109, USA

## Abstract

Memristors have emerged as a promising candidate for critical applications such as non-volatile memory as well as non-Von Neumann computing architectures based on neuromorphic and machine learning systems. In this study, we demonstrate that memristors can be used to perform principal component analysis (PCA), an important technique for machine learning and data feature learning. The conductance changes of memristors in response to voltage pulses are studied and modeled with an internal state variable to trace the analog behavior of the device. Unsupervised, online learning is achieved in a memristor crossbar using Sanger’s learning rule, a derivative of Hebb’s rule, to obtain the principal components. The details of weights evolution during training is investigated over learning epochs as a function of training parameters. The effects of device non-uniformity on the PCA network performance are further analyzed. We show that the memristor-based PCA network is capable of linearly separating distinct classes from sensory data with high clarification success of 97.6% even in the presence of large device variations.

The von Neumann architecture, widely used in conventional computing systems, has become less optimal in data-intensive tasks due to limited data transfer rates between the memory and the central processing unit. Alternative computing systems such as neuromorphic or machine learning systems, have attracted increasing attention in dealing with “big data” problems such as pattern recognition from large amounts of data sets^1^,^2^. Principal component analysis^3^ is an important technique used in machine learning to discover orthogonal factors underlying multivariate data by examining the correlations among the set of input variables. The technique can also be used to reduce the dimensionality of input data and is thus an important preprocessing step for many machine learning algorithms. Here we show that principal component analysis (PCA) can be efficiently achieved in simple memristor-based crossbar networks with online learning capability, allowing this technique to be used to effectively classify sensory data.

The two key factors that make memristor crossbar arrays attractive for neuromorphic or machine learning systems are 1) their ability to naturally implement matrix operations (*e.g.* dot-product): due to the resistive nature of the two-terminal device, the memristor crossbar array can directly convert an input voltage vector into an output current (or charge) vector, weighed by the memristor conductance at each matrix element, thus directly and efficiently performing the matrix operation; and 2) their ability to achieve online learning with simple programming pulses: the weights of the memristor crossbar matrix - the device conductances, can be incrementally trained using simple voltage pulses^4^,^5^. Other properties such as high density, low power consumption, long cycling endurance and subnanosecond switching speed have also been demonstrated in memristor devices^6^,^7^,^8^,^9^,^10^. A typical memristor device consists of a transition metal oxide layer such as TiO_x_, HfO_x_, WO_x_ sandwiched by a pair of electrodes^11^,^12^,^13^. The resistance of the memristor device can be adjusted by controlling the amount and distribution of oxygen vacancies, which modulate the local conductivity in the oxide layer^14^,^15^. Using an unsupervised, online learning rule, we show that crossbar arrays of memristors can learn the principal components from sensory data (e.g. database of breast cancer measurements) and effectively separate unlabeled data into clusters. After data clustering, a conventional supervised learning process can then be used to define a decision boundary and effectively classify tumors as malignant or benign.

## Results

### Memristor Behavior

**Figure 1:**
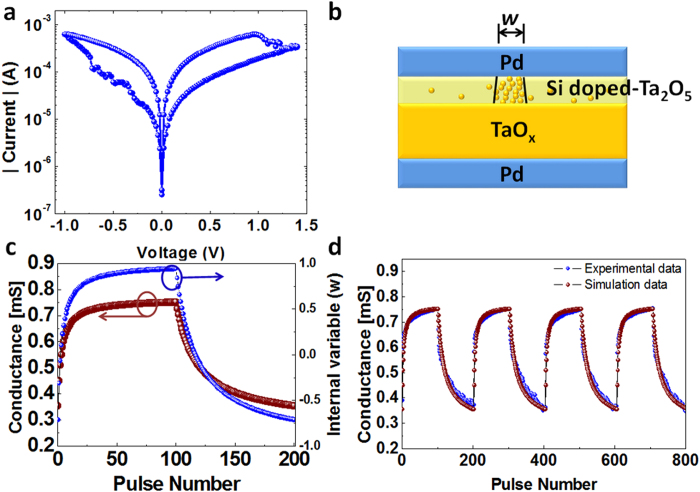
Memristor device and modeling. (**a**) DC I-V characteristics of a typical memristor device showing the bipolar switching effect. (**b**) Schematic of a memristor device. The region with high oxygen vacancy concentration (bounded by the black lines) forms the conduction channel. (**c**) Calculated memristor conductance and the internal state variable *w* during the application of 100 potentiation pulses (−1 V, 10 μs) and 100 depression pulses (1.15 V, 10 μs). (**d**) Measured (blue) and calculated (purple) conductance values measured by a read (0.2 V) pulse during 4 periods of 100 potentiation and 100 depression pulses.

The analog switching behavior is obtained from a tantalum-oxide memristor based on a bilayer structure consisting of an oxygen-rich Ta_2_O_5_ layer and an oxygen-deficient TaO_x_ layer^6^,^10^,^14^,^16^. We have shown that such a memristor with the tantalum oxide layer doped with silicon atoms can show improved dynamic range and controllable analog switching behavior^17^. In this study, 2 μm × 2 μm devices and crossbar arrays were used following the processes discussed in Ref. 17. During measurements, the bias voltage was applied to the top electrode (TE) while the bottom electrode (BE) was grounded. Fig. 1a shows DC current – voltage (I- V) curve of a device showing typical bipolar resistive switching characteristics. In this system, an applied voltage can change the amount and distribution of oxygen vacancies and modulate the conductive channels in the Ta_2_O_5_ layer which controls the conductance of the device^14^,^15^,^16^,^17^, as schematically shown in Fig. 1b.

To model the conductance change of the memristor, we introduce the internal state variable, *w*, which serves as an area index representing the number of conductive filaments or, equivalently, the area covered by the conductive channel as shown in Fig. 1b. The dynamics of the state variable in response to the applied voltage is described by equation (1), where *u*() is the Heaviside step function, *k, μ*_*1*_*, u*_*2*_, are positive parameters determined by material properties such as ion hopping distance and hopping barrier heights^13^ (Supplementary Information).


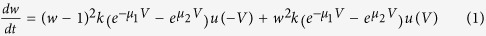



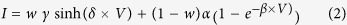


The current through the device is described by equation (2) which consists of the term describing conduction through the channel area (first term) and the rest of the device (Schottky-dominated conduction, second term)^13^. This equation clearly shows how the device conductance is regulated by the state variable, *w*. *γ, δ, α, β* are positive parameters determined by material properties such as the effective tunneling distance, tunneling barrier, the depletion width of the Schottky barrier region and Schottky barrier height^13^ (Supplementary Information). The memristor model, consisting of the state variable dynamic equation (1) and *I-V* equation (2), was tested against experimental measurements. For example, in Fig. 1c, pulse programming conditions were simulated with the application of a train of one-hundred −1 V, 10 μs pulses followed by a train of one-hundred 1.15 V, 10 μs pulses, with the device conductance monitored with a 0.2 V read pulse after each training pulse. With the application of a negative pulse, the memristor conductance gradually increases (purple curve), followed by the increase in the internal state variable value (blue curve). On the other hand, a positive pulse decreases the conductance following the decrease of the internal state variable value. The experimental data measured form an actual memristor device and the simulation data were compared and plotted together in Fig. 1d, showing that the model can trace the experimental data precisely.

### Neural Network Construction

**Figure 2:**
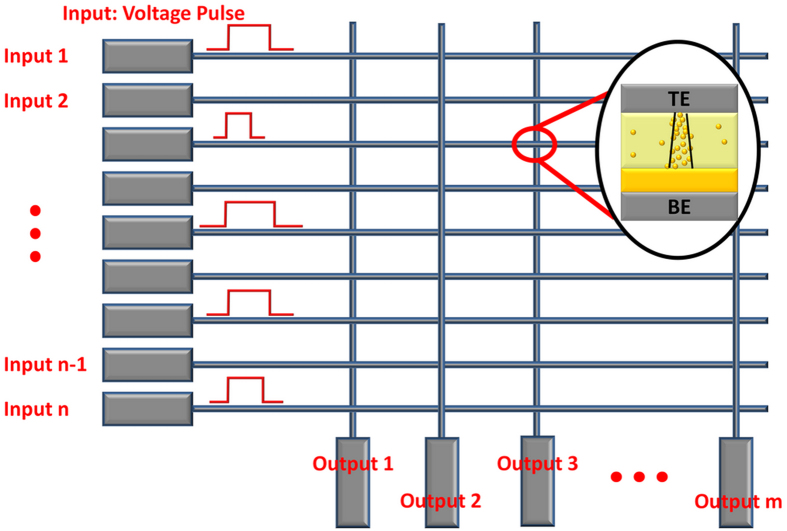
Schematic of the memristor network. The inputs are connected to the rows and fed to the network. The outputs are connected to the columns. The memristor devices are located at the crosspoints in the network and the weights of the memristor devices associated with a given output form the principal components after training.

To implement PCA, we adopted a neural network structure using a crossbar array of memristors as shown in Fig. 2, where the *n* input channels are connected to the rows and the *m* output channels are connected to the columns of the memristor crossbar network. In this study, a standard breast cancer data set from University of Wisconsin Hospitals, Madison was used as the input signal data^18^,^19^. The data set consists of breast cell mass properties in 9 categories including clump thickness, uniformity of cell size, uniformity of cell shape, marginal adhesion, single epithelial cell size, bare nuclei, bland chromatin, normal nucleoli and mitoses. The sensory data were derived from a digitized image of a fine needle aspirate (FNA) of a breast mass and each category has a range from 0 to 10. In a feature learning test, the measurement results from the 9 categories of a given cell are fed to the 9 inputs (*n* = 9) of the neural network, and the output is obtained from the 2 output channels (*m* = 2). The input signals are implemented as voltage pulses with fixed amplitude (0.2 V) and variable pulse widths proportional to the measured values in the corresponding category. Each training cycle consists of one hundred randomly sequenced data points (50 points from benign class, 50 points from malignant class). Afterwards, the ability of the network to successfully cluster the data and classify a cell as either benign or malignant was tested using 583 data points (not included in the training set).

As discussed earlier, in this configuration the output vector is determined by the dot-product of the input vector and the memristor weight matrix. Additionally, the network learns the principal components by adjusting the memristor weights during training. In this study, starting from a memristor network with randomly distributed weights, we employ Sanger’s rule (also known as the generalized Hebbian algorithm) to implement online learning to learn the principal components of the input data set. Sanger’s rule is derived from Hebb’s learning rule^20^,^21^ and these model learning rules have been widely adapted in artificial neural networks. Specifically, Sanger’s rule utilizes the weight (*g*), output response (*y*) and present input (*x*) as shown in equation (3).


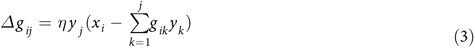


where η is the learning rate and is typically a small positive value(<<1), 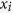 represents the input pulse at input (row) *i* and the value of the data is represented by the pulse width, and *j* = 1 or 2 corresponds to the primary principal component and the second principal component, respectively. *g*_*ij*_ is the weight at row *i* and column *j* in the network. Specifically, *g*_*ij*_ is defined as


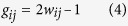


where *w*_*ij*_ is the state variable of the memristor device at row *i* and column *j* as discussed in Eq. (1). While *w* is positive only *g*_*ij*_ ranges from −1 to 1 from the definition. Note no label is used in the learning process. After training, the weights in columns 1 and 2 form the (first and 2^nd^, respectively) principal components of the input data set^21^. Accordingly, outputs obtained from the trained network will be clustered and can be used in subsequent classification analysis.

Specifically, with the application of an input *x*_*j*_, the amount of charge collected at the output in the memristor network can be obtained as:


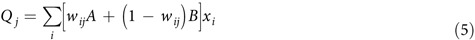


where the charge is assumed to be determined by the current (Eq. (2)) and linearly proportional to the applied pulse width (*x*_*i*_), and the constants in Eq. (2) have been lumped into constants A and B. The output, *y*_*j,*_ is then obtained from the charge *Q*_*j*_ through the following equation:


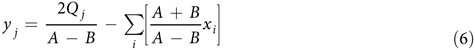


Plugging Eqs. (4, 5, 6) can be simplified as:


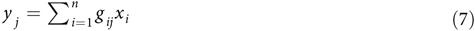


As expected, by properly choosing the output function (here linearly dependent on the charge, Eq. 6), the obtained output *y* corresponds to the vector product of the input and the weight matrix, as required by neural network algorithms.

During the training phase, the output is first obtained (by applying a 0.2 V read voltage with a pulse width proportional to the value of the training data at each column) from the memristor array using equation (6), and the desired weight update 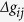 is then calculated based on equation (3). Programming voltage pulses are then applied to the inputs to modify the memristor weights. The programming pulses are determined by the polarity and magnitude of 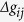, with potentiation (−1 V) pulses applied to the input for positive 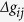 and depression (1.15 V) pulses for negative 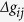, while the pulse widths are determined by the magnitude of 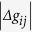. To account for the non-linear response of *w* with respect to training pulse (*i.e.* the effectiveness of weight change *dw*/*dt* depends on the device state *w*, as evidenced in Eq. 1 and Fig. 1c-d), a compensation scheme is employed to ensure the desired conductance change. Specifically, the pulse width 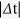 is determined as


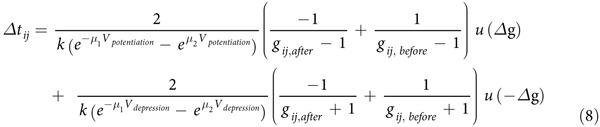


When applied to equation (1) and by noticing the relationship between *w* and *g* (eq. 4), equation (8) leads to the desired weight change in equation (3).

**Figure 3:**
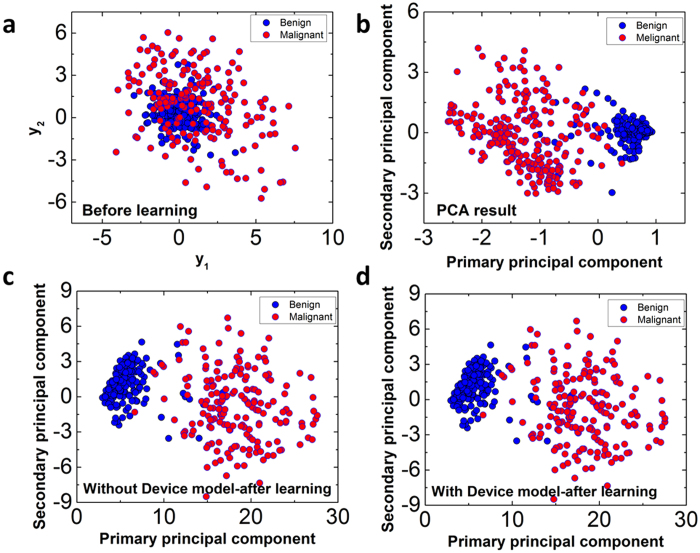
Results of principal component analysis. (**a**) Initial results of an untrained network. The data are plotted based on their (y_1_,y_2_) values. Linear separation is not possible for the two classes. (**b**) Principal component analysis using traditional covariance matrix of the input data. (**c**) Principal component analysis using Sanger’s rule. (**d**) Principal component analysis using Sanger’s rule with the memristor physical device model.

Figure 3a shows results of the 583 test data points before learning (*e.g.* when the memristor weights are random), with y_1_ at horizontal axis and y_2_ at vertical axis. Blue dots and purple dots represent benign and malignant cells (the ground truth), respectively. We note the labels were not used during training and are only shown here to illustrate the effectiveness of the clustering process. It’s clear from Fig. 3a that before training the benign set and the malignant set significantly overlap each other. In other words, the network before learning cannot effectively cluster the sets (with untrained, random weights). Results obtained after performing classical PCA calculations by directly calculating the eigenvectors and eigenvalues of the data covariance using matrix operations are shown in Fig. 3b. The PCA calculations perform orthogonal transformation to identify the primary principal component in the direction of the largest variance, and subsequently the 2^nd^ principal component, etc^3^. As expected, the data become clustered after transforming the data along the first two principal components, as shown in Fig. 3b. Instead of directly calculating the principal components using matrix operations and existing data, the principal components can also be obtained through training in neural networks, as discussed earlier. Figure 3c shows results obtained from an idealized neural network using Sanger’s rule, using only equation (3) and equation (7) without considering the physical memristor device model. Successful clustering of the data set was also achieved in the neural network^21^. In this case, instead of computed from current data set, the principal components were learned using Sanger’s rule and are represented by the weights associated with specific outputs. More importantly, Fig. 3d shows the results obtained in the neural network employing the physical memristor device model during training and feature extraction analysis. Successful clustering of the data, similar to the ones obtained from direct PCA calculations and learning with an ideal neural work, was also obtained in the memristor network, suggesting the potential of the memristor networks for feature learning tasks with online, unsupervised learning.

**Figure 4:**
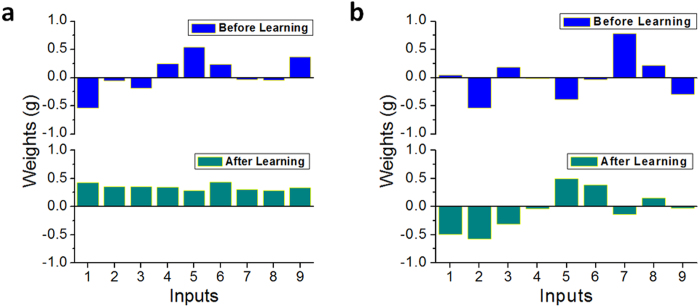
Weights constituting (**a**) the primary principal component and (**b**) the secondary principal component before (upper) and after (lower) the learning process.

Figure 4 shows the primary and secondary principal components learned in the memristor network from the training process, represented by the two 9-dimentional weight vectors associated with the two outputs. The training consists of 1000 training cycles. Since the application of Sanger’s rule automatically normalizes the weights the Euclidean norm of the weights should converge to 1 after training (Supplementary Information). Indeed, the length of the weight vector for the primary principal component was found to converge from 0.9 to 1.0005 and that for the secondary principal component was found to converge from 1.12 to 1.003. In practice, this normalization condition can be used to determine when the network has completed learning.

**Figure 5:**
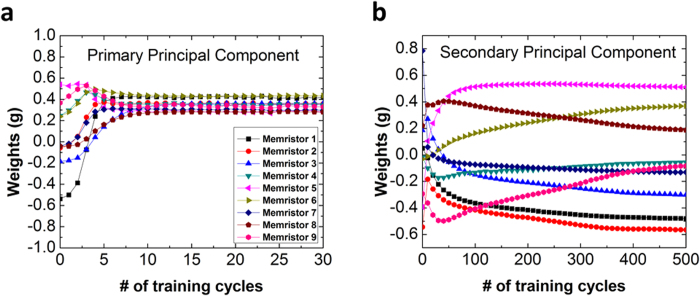
Weight change as a function of training cycles for (**a**) the primary principal component, (**b**) the secondary principal component.

To examine how the weights change during learning, weight distributions for the first two principal components during training are plotted in Fig. 5. For the primary principal component (Fig. 5a), the weights change rapidly in the first 10 cycles and quickly become stabilized for the rest of the learning cycles. While for the secondary principal component (Fig. 5b) the weights change gradually and the distribution stabilizes at a much later time. The reason for the different behaviors lie in the fact that for the primary principal component, only y_1_ and g_i1_ need to be taken into account during weight update (equation (3)); however, for the secondary principal component, both y_1_, y_2_, and g_i1_ and g_i2_ need to be considered so convergence of the secondary principal component is more difficult and only happens after the primary principal component has stabilized.

**Figure 6:**
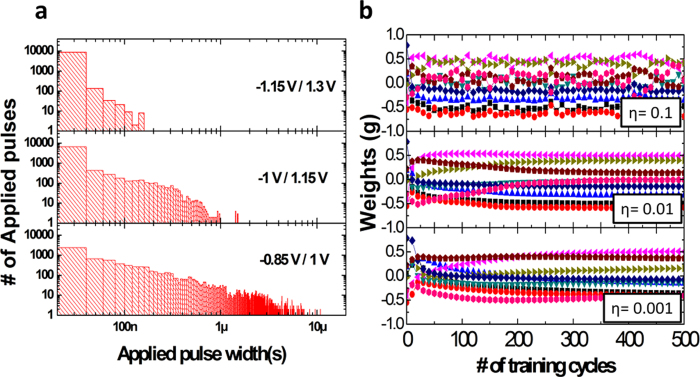
Effects of potentiation/depression voltage amplitudes and learning rate. (**a**) Histograms of the applied pulse widths used in training as a function of potentiation/depression voltage amplitude. (**b**) The weight evolutions as a function of learning rate.

The effect of the applied voltage during learning and the learning rate are shown in Fig. 6. Figure 6a shows the histogram graphs of the number of pulses used during the training processes for different pulse amplitudes, measured in 20 ns intervals. As expected, it can be seen that lower potentiation/depression voltages requires longer pulse widths in general, while faster learning can be obtained at higher voltages. Additionally, Fig. 6b shows the effect of the learning rate, η, on the training process. The weight redistribution for the secondary principal component as a function of training is plotted. If the learning rate is too high (η=0.1), weight update becomes too fast (Eq. 3) and can overshoot the optimal value. As a result, the weight distributions fluctuate during training and never fully stabilize, as shown in the top graph in Fig. 6b. On the other hand, if the learning rate is too small (η=0.001), the weight updates becomes very slow and may not be able to overcome local minima, as shown in the bottom graph in Fig. 6b. A properly chosen learning rate (η=0.01) balances learning speed and accuracy.

**Figure 7:**
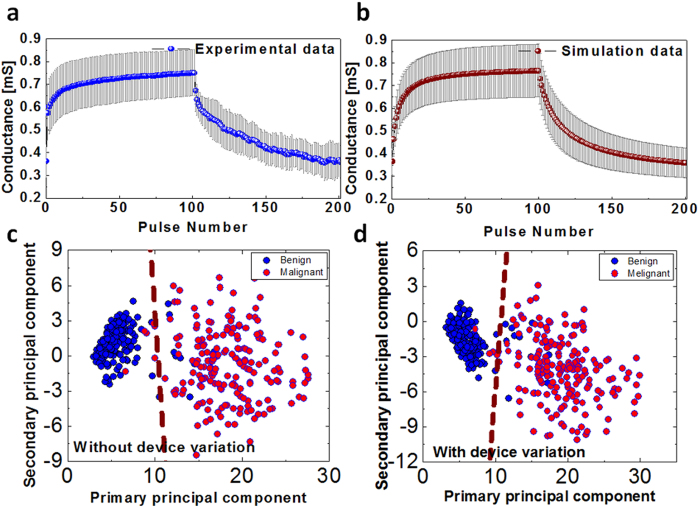
Effects of device variations. (**a**) Experimentally measured analog switching data from 9 memristors during 100 potentiation and 100 depression pulses. The blue line and the error bars represent the average and the standard deviation, respectively. (**b**) Calculated analog switching behaviors after considering device variations in the model. (**c**) Results of the principal component analysis without device variability. (**d**) Result of the principal component analysis with realistic device variability captured by the model.

In the following, we discuss the effects of device-device variations in the network performance. Nanoscale devices such as memristors whose operations are essentially based on defects (e.g. oxygen vacancies) are intrinsically less reliable than conventional transistor devices. As shown in Fig. S1a and Fig. S1b, large device-device and cycle-cycle variations exist in the analog switching behaviors of memristors. The variations in the memristor switching characteristics can be attributed to variations in device parameters such as the amount and distribution of oxygen vacancies in the conduction channel area, resistance variations of the TaO_x_ base region, stoichiometric non-uniformity and film thickness variations. Figure 7a shows the conductance changes of 9 memristor devices in the network during the application of 100 pulses of potentiation (−1 V) and 100 pulses of depression (1.15 V). The blue line represents the average value and the error bars represent the standard deviation of the measured conductance. The relative standard deviation ranges from 10% to 23% for each point and are clearly substantial. To understand the effects of the device variations on the network performance, variations were introduced to the physical device parameters in Eqs. (1)-(2), 2, and simulation results after incorporation of device variations are shown in Fig. 7b, capturing the same average value and standard deviation as the measured data. Details of the measured data and modeling can be found in the Supporting Information. The learning and PCA classification results of the memristor network, with and without considering device variations, are shown in Fig. 7c and 7d for comparison. Significantly, even with substantial device-device and cycle-cycle variations (Fig. 7b), the network is still able to successfully learn the principal components and classify the data sets into the 2 categories (Fig. 7d). The training becomes slightly less optimal with the length of the weight vectors increased slightly to 1.05 and 1.06 for the primary and secondary principal components, respectively, compared to 1.0005 and 1.003 without considering device variations.

Finally, to quantitatively analyze the performance of the memristor network, logistic regression^22^ was used to analyze the clustered data to measure the effectiveness of the PCA analysis. The linear decision boundaries obtained from logistic regression are shown as dotted lines separating the two clustered sets of data in Fig. 7c and 7d. Classification based on linear decision boundaries on the clustered data obtained from different approaches yielded essentially identical results (97.4% in Fig. 7c for the ideal case without considering device variations, and 97.6% in Fig. 7d for the case considering realistic device variations). This result suggests that the memristor network can be inherently tolerant to device variations due to the distributed network structure, and systems based on such networks can lead to reliable operations despite the nanoscale devices being intrinsically unreliable.

## Discussion

In conclusion, we show that memristor networks can effectively implement unsupervised learning rules and be trained to learn principal components from data sets. The principal components learned during the training process can then be directly used to perform feature extraction (clustering) tasks using the same network. A realistic physical model was developed for the TaOx based memristor and used in the analysis. Sanger’s learning rule was utilized to implement online learning by adjusting the weights of each memristor in the crossbar network. After learning the principal components, the memristor network was successfully used to classify breast cancer data set as an example through first data clustering and then deriving a linear decision boundary. Significantly, successful learning and classification can still be obtained in the memristor network even in the presence of substantial device variations, demonstrating the reliability of the network structure and the learning algorithm. The ability to achieve online learning and perform classification tasks reliably in the presence of unreliable devices suggest this approach can be extended to larger networks and other machine learning algorithms for more complex data-intensive tasks.

## Methods

### Device fabrication and characterization

The device fabrication starts with a highly p-doped Si/SiO_2_ substrate with a 100 nm thermal SiO_2_ layer. The bottom electrodes (BEs) were fabricated by photolithography and liffoff with 5nm-thick NiCr and 40nm-thick Pd. The 40 nm of oxygen-rich TaO_x_ layer was sputtered by direct current (DC) reactive using a Ta metal target with Ar(97%)/O_2_(3%) gas mixture at 400 °C. Next, 5 nm of Ta_2_O_5_ switching layer was sputtered by 140 W radio frequency (RF) sputtering while p-doped Si was co-sputtered with Ta_2_O_5_ layer with 70 W DC sputtering at room temperature. The top electrodes (TEs) with 40 nm of Pd and 20 nm of Au were fabricated by photolithography and liffoff to form a crossbar structure. The electrical characterization were performed with a custom-built measurement system in a probe station (Desert Cryogenics TTP4).

### Simulation

Device model fidelity was verified using SPICE simulations and then translated to Python for network simulation integration. Array scale simulations were performed in a custom, multithreaded framework developed in Python. The framework makes extensive use of the NumPy module^23^ for optimized calculations and the Matplotlib module^24^ for data visualization.

## Additional Information

**How to cite this article**: Choi, S. *et al*. Data Clustering using Memristor Networks. *Sci. Rep.* **5**, 10492; doi: 10.1038/srep10492 (2015).

## Supplementary Material

Supplementary Information
