# Supplementary material for: Data Clustering using Memristor Networks
Source: Sci Rep. 2015 May 28;5:10492. doi: 10.1038/srep10492 (PMC4650685; doi:10.1038/srep10492)
Supplement: Supplementary Information [file srep10492-s1.pdf]

<Supplementary Information>

Data Clustering Using Memristor Networks

Shinhyun Choi[1], Patrick Sheridan[1] & Wei D. Lu[1,*]

[1]Department of Electrical Engineering and Computer Science, University of Michigan, Ann Arbor, Michigan 48109, USA

* Correspondence to W.D.L. (email: wluee@eecs.umich.edu).

1. Device Modeling

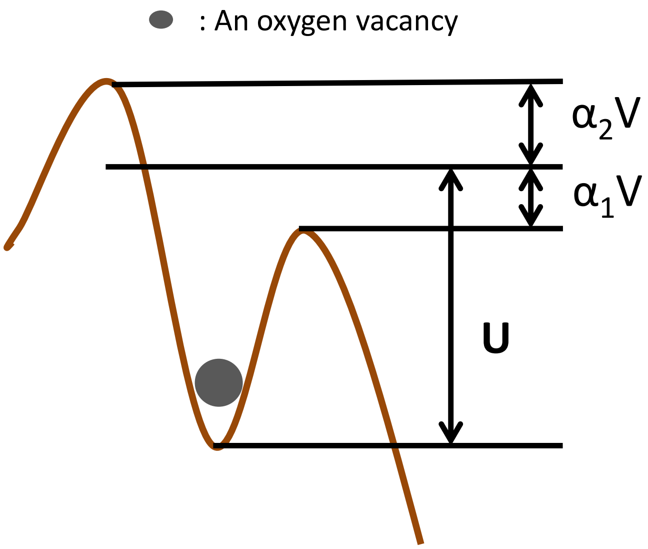

Figure S1. Energy barrier of ion hopping process.

The growth rate of the state variable, *w*, is determined by ion hopping process over an energy barrier as shown in figure S1. It can be written as [S1- S3]:

$$\frac{\mathrm{dw}}{\mathrm{dt}} = f(w, V) \cdot \mathrm{d} \cdot \mathrm{f}\left[\exp\left(\frac{-\mathrm{q}(\mathrm{U}-\alpha_1 V)}{\mathrm{kT}}\right) - \exp\left(\frac{-\mathrm{q}(\mathrm{U}+\alpha_2 V)}{\mathrm{kT}}\right)\right] \quad \text{(S1)}$$

where *d* is half of the average hopping distance of ions, *f* is the attempt frequency, *q* is the charge of an electron, *U* is the activation potential energy, *k* is the Boltzmann's constant, *T* is the temperature in Kelvin, α_1 and α_2 are barrier lowering coefficients [S1], and *f(w, V)* is a window

function to account for the non-linear response to the applied voltage [S2]. The window function used in this paper is shown in equation S2:

$$f(w, V) = (w - 1)^2 u(-V) + w^2 u(V) \qquad \text{(S2)}$$

Where *u()* is the Heaviside step function. By plugging equation (S3) into equation (S1), the rate equation of w can be re-written as:

$$\frac{dw}{dt} = (w - 1)^2 k(e^{-\mu_1 V} - e^{\mu_2 V})u(-V) + w^2 k(e^{-\mu_1 V} - e^{\mu_2 V})u(V) \qquad \text{(S3)}$$

where $k = df\ exp(\frac{-qU}{kT})$, $\mu_1 = \exp(\frac{-q\alpha_1}{kT})$, and $\mu_2 = exp(\frac{-q\alpha_2}{kT})$. Eq. (S3) is Eq. (1) in the main text.

The current through the device described by equation (2) consists of tunneling-dominated conduction and Schottky-dominated conduction. The tunneling current can be calculated by assuming MIM structure with very thin insulator so that the tunneling current is observed. Using the expressions for a square barrier [S4, S5, S6], the current can be derived as:

$$I = A\frac{4q\pi m(kT)^2}{h^3}\exp(-b_1)\frac{1}{(c_1 kT)^2}\frac{\pi c_1 kT}{\sin(\pi c_1 kT)}(1 - \exp(c_1 qV)) \qquad \text{(S4)}$$

Where

$$b_1 = \frac{2\alpha d\sqrt{q}}{3V}({\varphi_0}^{\frac{3}{2}} - (\varphi_0 - V)^{\frac{3}{2}}) \quad \text{if } V < \varphi_O \qquad \text{(S5)}$$

$$= \frac{2\alpha d\sqrt{q}}{3V}({\varphi_0}^{\frac{3}{2}}) \qquad \text{if } V > \varphi_O$$

$$c_1 = \frac{\alpha d}{V\sqrt{q}}({\varphi_0}^{\frac{1}{2}} - (\varphi_0 - V)^{\frac{1}{2}}) \quad \text{if } V < \varphi_O \qquad \text{(S6)}$$

$$= \frac{\alpha d}{V\sqrt{q}}(\varphi_0{}^{\frac{1}{2}}) \qquad\qquad \text{if V} > \varphi_O$$

A is the filament area, m is the effective electron mass, h is Plank's constant, φ_0 is the barrier height at zero bias, d is the tunneling distance, and $\alpha = \frac{4\pi\sqrt{2m}}{h}$.

At low bias, equation (S5) and (S6) can be simplified as:

$$b_1 = \frac{2\alpha d\sqrt{q}}{3V}\left(\varphi_0{}^{\frac{3}{2}} - (\varphi_0 - V)^{\frac{3}{2}}\right)$$

$$= \frac{2\alpha d\sqrt{q}}{3V}\varphi_O{}^{\frac{3}{2}}\left(1 - \left(1 - \frac{V}{\varphi_0}\right)^{\frac{3}{2}}\right)$$

$$= \frac{2\alpha d\sqrt{q}}{3V}\varphi_O{}^{\frac{3}{2}}(1 - \left(1 - \frac{3V}{2\varphi_0}\right))$$

$$= \frac{2\alpha d\sqrt{q}}{3V}\varphi_O{}^{\frac{3}{2}}\left(\frac{3V}{2\varphi_0}\right) \qquad \text{(S7)}$$

$$c_1 = \frac{\alpha d}{2\sqrt{q\varphi_0}} \qquad \text{(S8)}$$

By plugging equation (S7) and equation (S8) into the equation (S4),

$$\text{I} = \text{A}\frac{4q\pi m(kT)^2}{h^3}\exp(-b1)\frac{1}{(c_1kT)^2}\frac{\pi c_1 kT}{\sin(\pi c_1 kT)}(1 - \exp(c_1 qV))$$

$$= \text{A}\frac{4q\pi m(kT)^2}{h^3}\frac{1}{(c_1kT)^2}\frac{\pi c_1 kT}{\sin(\pi c_1 kT)}e^{-\alpha d\sqrt{q\varphi_0}}e^{\frac{\alpha dV}{4}\sqrt{\frac{q}{\varphi_0}}}\left(1 - e^{\frac{-\alpha dV}{2}\sqrt{\frac{q}{\varphi_0}}}\right)$$

$$= \mathrm{A}\frac{4q\pi m(kT)^2}{h^3}\frac{1}{(c_1kT)^2}\frac{\pi c_1 kT}{\sin(\pi c_1 kT)}e^{-\alpha d\sqrt{q\varphi_0}}\sinh(\frac{\alpha dV}{4}\sqrt{\frac{q}{\varphi_0}})$$

$$= \mathrm{A}\frac{16kT\pi^2 mq\sqrt{q\varphi_0}}{\alpha dh^3\sin\left(\frac{\pi\alpha dkT}{2\sqrt{q\varphi_0}}\right)}e^{-\alpha d\sqrt{q\varphi_0}}\sinh(\frac{\alpha dV}{4}\sqrt{\frac{q}{\varphi_0}}) \quad \text{(S9)}$$

For the Schottky junction current is explained by equation (S10).

$$I = \frac{qADn}{L}\left(1 - exp\left(-\frac{qV}{\vartheta kT}\right)\right) \quad \text{(S10)}$$

where q in the charge of an electron, A is the dimension of the device, D is the diffusion coefficient, n is the number of electrons, L is the diffusion length of electrons, V is the applied voltage, ϑ is an ideality factor, k is the Boltzmann's constant and T is the temperature in Kelvin.

The total current includes the parallel contribution from the filament region (with relative area w) and Schottky region (with relative area 1-w). Combining Eqs. (S9) and (S10), one obtains:

$$I = w\,\gamma\sinh(\delta \times V) + (1 - w)\alpha\left(1 - e^{-\beta\times V)}\right) \quad \text{(S11)}$$

Where γ, δ, α, β corresponds to the parameters defined in (S9) and (S10).

2. Details of the measured data and modelling

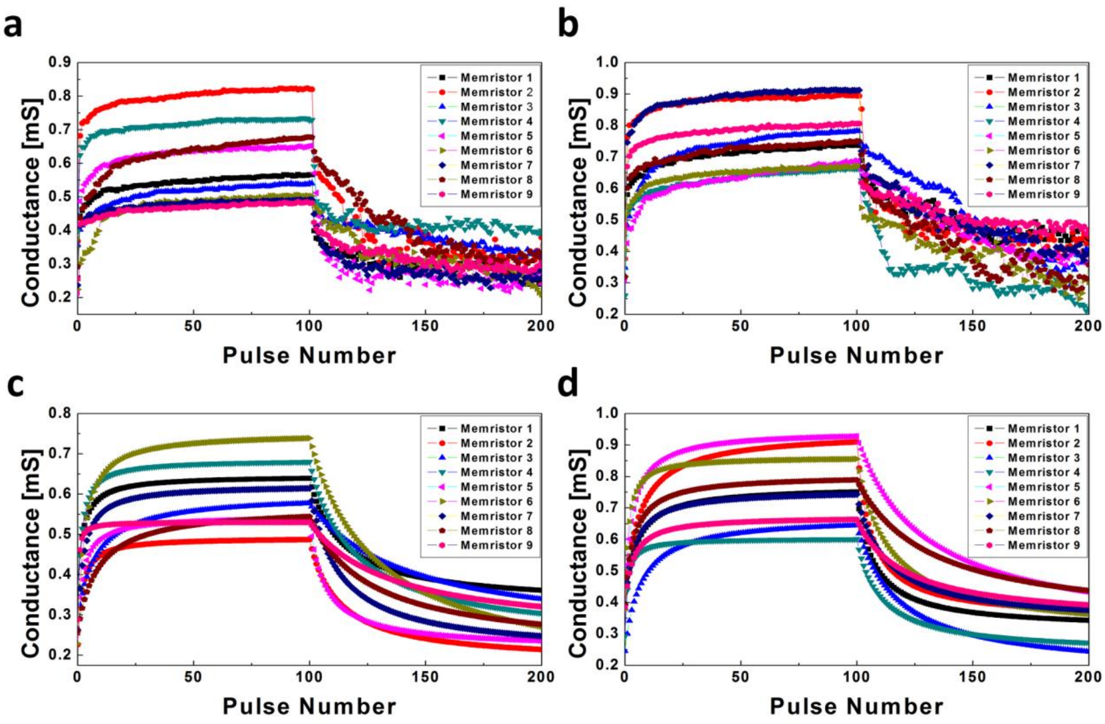

Figure S2. Experimental measurements on the memristor devices in a network showing the analog conductance change and device-device variations. The conductance was measured with 0.2 V, 1 ms pulses, and the devices were subject to 100 pulses of potentiation (-1 V, 10 μs) and 100 pulses of depression (1.15 V, 10 μs). (a, b) experimental results for the 9 memristors corresponding to the primary principal component (a) and the 9 memristors corresponding to the second principal component (b). (c, d) modeled conductance for the cases in (a, b) using equations (1) and (2).

Figure S2 shows the analog conductance changes of the 18 memristor devices forming the network. Device-device variations are clearly observed which causes conductance discrepancy

after potentiation/depression pulses. To verify the effect of device variations on the network performance, the relevant device parameters were assumed to vary following Gaussian distributions (Table S1) and the exact value of a parameter for a given device was chosen randomly using a Monte Carlo method during the simulations. Figure 7b shows the average value and standard deviation calculated using this approach, which are consistent with the experimentally observed variations. The model with the random device variations was then applied to the network analysis and led to Figure 7d. The parameters used in the simulation are shown in Table S1.

Variable	Primary Principal Component		Secondary Principal Component	
	Nominal value	σ/(Nominal value)	Nominal value	σ/(Nominal value)
κ	6×10^{-5}	3%	6×10^{-5}	3%
η_1	16	3%	16	3%
η_2	20.2	3%	20.2	3%
α	5×10^{-4}	15%	6×10^{-4}	15%
β	0.5	3%	0.5	3%
γ	2×10^{-3}	10%	2.55×10^{-3}	10%
δ	0.3	3%	0.3	3%

Table S1. Device parameters used in the simulation.

3. Weight normalization with Sanger's Rule

The Sanger's rule originates from Hebb's rule [S7]. For the simplicity, with the assumption that we have only one output, we can write the Hebb's rule as shown in the equation below.

$$w_i(n+1) = w_i + \eta y x_i \tag{S12}$$

If we require the weights to be normalized to prevent infinite growing output of Hebb's rule, the update rule is modified as:

$$w_i(n+1) = \frac{w_i + \eta y x_i}{\sqrt[2]{\sum_{j=1}^{m}(w_j + \eta y x_j)^2}} \tag{S13}$$

where m is the number of inputs. Because η, the update rate, is normally very small(<<1), through Taylor expansion and keeping only the leading term, (S13) becomes.

$$w_i(n+1) = w_i + \eta y(x_{i-}w_i y) \tag{S14}$$

Eq. (S14) is the equation for Sanger's rule (with only 1 output. The case for more than one outputs can be derived similarly). In other words, by implementing Sanger's rule (S14), we are effectively implementing rule (S13) (again for small update rates η which is satisfied in experiments) with normalized weights.

REFERENCES

(S1) Ielmini, D. Modeling the Universal Set/Reset Characteristics of Bipolar RRAM by Field- and Temperature- Driven Filament Growth. *IEEE Trans. Elec. Dev.* **58,** 4309–4317 (2011).

(S2) Chang, T., Jo, S., Kim, K.H., Sheridan, P., Gaba, S. & Lu, W. Synaptic behaviors and modeling of a metal oxide memristive device. *Appl. Phys. A*, **102**, 857-63 (2011).

(S3) Kim, S., Choi, S. & Lu, W. Comprehensive physical model of dynamic resistive switching in an oxide memristor. *ACS Nano* **8,** 2369–76 (2014).

(S4) Stratton, R. Volt-current characteristics for tunneling through insulating films. *J. Phys. Chem. Solids* **23**, 1177-90 (1962)

(S5) Simmons, J. G. Generalized Formula for the Electric Tunnel Effect between Similar Electrodes Separated by a Thin Insulating Film. *J. Appl. Phys.* **34**, 1793-1803 (1963).

(S6) Yang, J. J. *et al.* Memristive switching mechanism for metal/oxide/metal nanodevices. *Nat. Nanotechnol.* **3,** 429–33 (2008).

(S7) Sanger, T. D. Optimal unsupervised learning in a single-layer linear feedforward neural network. *Neural Networks*, **2**, 459-73 (1989).

Disclaimer: The views expressed here are those of the author and do not reflect the official policy or position of the Department of Defense or the U.S. Government. Approved for Public Release. Distribution unlimited.
